# Die Soziale Marktwirtschaft nach der Corona-Krise: Fit für den Systemwettbewerb im 21. Jahrhundert

**DOI:** 10.1007/s41025-021-00226-3

**Published:** 2021-06-15

**Authors:** Christoph M. Schmidt

**Affiliations:** RWI – Leibniz-Institut für Wirtschaftsforschung, Essen, Deutschland

**Keywords:** Systemwettbewerb, Nachhaltiges Wachstum, Globale Kooperation, Regeltreue, Technologietransfer, Systems competition, Sustainable growth, Global cooperation, Compliance, Technology transfer

## Abstract

Die Soziale Marktwirtschaft ist auch in Zeiten des verschärften Systemwettbewerbs mit den USA und China die geeignete Wirtschafts- und Gesellschaftsordnung für Deutschland und Europa, um die Volkswirtschaft auf einen nachhaltigen Wachstumspfad zu führen. Nur ein wirtschaftlich erfolgreiches Europa wird erfolgreich dabei mitwirken, das 21. Jahrhundert auf Basis der eigenen Werte als eine Ära der weltumspannenden materiellen Prosperität und ökologischen Nachhaltigkeit zu gestalten. Daher gilt es vor allem, die europäische Klimapolitik marktwirtschaftlich auszugestalten und die eigene Regeltreue und Vorbildlichkeit bei der Umsetzung globaler Vertragswerke mit hoher Bereitschaft zum Finanz- und Technologietransfer zu verbinden.

## Abstract (en)

In times of a tightened competition of systems, being sandwiched by China and the US, the concept of a social market economy still provides the best approach for Germany and Europa to ascertain sustainable economic growth. Only if Europe is economically successful, it will be able to contribute on the basis of its own values to shaping the 21st century as an era of global material prosperity and ecological sustainability. Consequently, Europe should pursue a market-oriented approach to climate policy and to combine strict compliance with global agreements with strong engagement into the transfer of funds and technologies.

## Deutschland und Europa im Systemwettbewerb

Nachdem rund ein Jahr seit dem Beginn der Corona-Pandemie vergangen ist, fällt das Zwischenfazit für das Krisenhandeln in Deutschland gemischt aus. So ist es gelungen, einen Zusammenbruch des Gesundheitssystems abzuwenden und den gesamtwirtschaftlichen Einbruch des Jahres 2020 zu begrenzen. Wenngleich die wirtschaftliche Erholung durch die zweite und dritte Infektionswelle gedämpft wurde (GD 2021), eröffnet das zunehmende Tempo bei der Nutzung der in atemberaubender Geschwindigkeit entwickelten Impfstoffe nun endlich die Aussicht auf ein Ende der akuten Krise noch im Verlauf des Jahres 2021.

Das sind bemerkenswerte Erfolge einer gewaltigen Kraftanstrengung von Gesellschaft, Wirtschaft, Politik und Wissenschaft. Während weltweit die absolute Armut entgegen dem Trend der vergangenen Jahrzehnte in der Pandemie drastisch zugenommen hat und damit Jahrzehnte des Fortschritts zunichte gemacht wurden, haben hierzulande die Systeme der sozialen Sicherung und Gesundheitsversorgung dafür gesorgt, dass inmitten der Krise der Anstieg sozialer Ungleichheit begrenzt blieb. Es zeigen sich allerdings auch für Deutschland zunehmend die Kollateralschäden der langanhaltenden Krise, etwa im Bildungsbereich (SVR 2020).

Nun stellt sich trotz der von den Impfstoffen versprochenen Entlastung die Frage, wie es in Deutschland und Europa weitergehen soll. Das betrifft zum einen den Ausstieg aus dem Krisenhandeln: Schon seit geraumer Zeit besteht die Herausforderung, eine neue Normalität des gesellschaftlichen und wirtschaftlichen Lebens mit dem Corona-Virus und seinen Mutationen zu gestalten (acatech 2020). Wenngleich Krisenmanagement und -kommunikation durch Bund und Länder anfänglich recht erfolgreich schienen, hat sich inzwischen ein intensiver Diskurs über das richtige Ausmaß staatlich gesetzter Freiheitsbeschränkungen entfaltet.

Zum anderen haben die weltumspannende Natur der Krise und der mit ihr verbundene Bedeutungsgewinn staatlichen Handelns die Frage aufgeworfen, welche Wirtschafts- und Gesellschaftsordnung unter den Bedingungen des 21. Jahrhundert angemessen ist. Neben der Anforderung, heimische Prosperität zu schützen, geht es vor allem um die Fähigkeit, die Wirtschaft künftig auf einen nachhaltigen Wachstumspfad zu führen. Europa steht dabei in einem scharfen Wettbewerb der Systeme, nicht zuletzt mit den USA und China, die gewillt sind, eigene Dominanzansprüche mit aktiver Industriepolitik durchzusetzen (SVR 2018, 2019b).

Im weltweiten Systemwettbewerb geht es zudem um weit mehr als die nationale Prosperität. Die weltwirtschaftliche Entwicklung der vergangenen drei Jahrzehnte ist zwar eine große Erfolgsgeschichte: Hunderte von Millionen Menschen weltweit wurden aus der Armut geführt. Dies ist vor allem durch eine vertiefte internationale Arbeitsteilung gelungen, die durch die abnehmende Bedeutung physischer Distanzen ermöglicht wurde und weit entfernt voneinander lebende Gesellschaften eng miteinander verknüpft hat. Allen voran hat dafür vor allem seit der Jahrhundertwende die Integration Chinas in den Welthandel gesorgt.

Doch gleichzeitig haben die weltweit wachsende durchschnittliche Wirtschaftsleistung pro Kopf und die unaufhörlich wachsenden Bevölkerungszahlen vor allem in Asien unseren Planeten an die Grenzen seiner Belastbarkeit geführt. In den kommenden drei Jahrzehnten muss es nun gelingen, auf dem friedlichem Wege der globalen Kooperation eine neue Balance zu finden. Sie muss hohen Respekt vor den Menschen – ihrem Streben nach einer besseren materiellen Zukunft, nach kreativer Entfaltung, Freiheit und Selbstbestimmung sowie nach einer fairen Verteilung der Lebenschancen – mit hohem Respekt vor der Natur verbinden.

Ein 21. Jahrhundert der weltumspannenden materiellen Prosperität und ökologischen Nachhaltigkeit erfordert die freiwillige und vertragstreue Kooperation ganz unterschiedlich organisierter Nationen. Dies herbeizuführen, allem voran bei der Klimapolitik, ist angesichts der Ausstrahlungseffekte der eigenen Handlungen auf die Lebensumstände anderer Gesellschaften eine riesige Herausforderung. Denn im globalen Kontext müssen anreizkompatible vertragliche Regelungen an die Stelle von gesetzlichen Regelungen treten, die nur innerhalb von Nationen für die Internalisierung externer Effekte sorgen können (Ockenfels und Schmidt 2019).

Im Mittelpunkt dieser Diskussion steht die Umstellung des globalen Energiesystems auf Klimaneutralität. Die Europäische Union (EU) hat sich vorgenommen, dabei eine Vorreiterrolle einzunehmen. Mit ihrer Strategie eines „European Green Deal“ will sie das Ziel der Klimaneutralität bereits im Jahr 2050 erreichen. Nun müssen die EU und ihre Mitgliedstaaten nicht nur dieses ambitionierte Ziel erreichen, sondern dabei auch wirtschaftliche Leistungsfähigkeit und sozialen Ausgleich bewahren. Nur so wird die EU die Klimapolitik tatsächlich auf der globalen Ebene prägen und so ihre eigenen Werte im Wettbewerb der Systeme durchsetzen können.

Der vorliegende Beitrag diskutiert diese doppelte Herausforderung an das deutsche und auch für das integrierte Europa prägende Gesellschafts- und Wirtschaftssystem, die Soziale Marktwirtschaft. Welches System ist künftig das richtige, um wirtschaftliche Prosperität und gesellschaftlichen Zusammenhalt in Deutschland und Europa sicherzustellen? Und wie kann es gelingen, auf dem Weg zur globalen Prosperität und ökologischen Nachhaltigkeit – hier verdichtet auf das Ziel der Klimaneutralität – im Wettbewerb der Systeme so zu bestehen, dass die europäischen Wertvorstellungen nicht unter die Räder geraten?

## Soziale Marktwirtschaft: das Referenzmodell in der Corona-Krise

Die Soziale Marktwirtschaft hat sich über Jahrzehnte als Wirtschafts- und Gesellschaftsordnung bewährt: Sie hat ein erhebliches Wohlstandswachstum ermöglicht und gleichzeitig für eine bemerkenswerte Kohärenz der Einkommensverteilung gesorgt sowie die Schwächsten in adversen Lebenslagen besonders geschützt. Dies ist bis zuletzt gelungen, obwohl mit der rasch eingeleiteten, wenngleich nicht vollständigen Heranführung der ostdeutschen Bundesländer an den westdeutschen Lebensstandard nach 1990 angesichts des faktischen Bankrotts der Wirtschaft der DDR eine gewaltige Integrationsleistung zu stemmen war (SVR 2019c).

Mit dieser Leistung ist die Soziale Marktwirtschaft – nicht nur in Europa – zum Referenzpunkt für eine effiziente und robuste Gesellschafts- und Wirtschaftsordnung geworden. Ihr zentrales Strukturprinzip besteht darin, marktwirtschaftliche Effizienz mit der Sicherstellung breiter Teilhabe am dadurch ermöglichten Wohlstand zu verbinden. Kernelemente effizienter Märkte sind die unternehmerische Freiheit, das durch das Haftungsprinzip gebundene Privateigentum und als Systemimperativ der durch staatliches Handeln gegen Machtkonzentrationen geschützte Wettbewerb (Empter und Esche 2018; Petersen und García Schmidt 2018).

Der soziale Ausgleich wird in seiner Wirkung zwar vorwiegend *ex post* im umfassenden Steuer- und Transfersystem sichtbar, aber der vielleicht noch wichtigere Baustein der sozialen Teilhabe besteht *ex ante* darin, durch breiten Zugang zur Mitwirkung am Wirtschaftsprozess die Möglichkeit zum sozialen Aufstieg zu eröffnen. Ob es nach wie vor gelingt, den hohen Anspruch der Chancengerechtigkeit zu erfüllen, wird zwar intensiv diskutiert (Fratzscher 2016; Peichl und Barišić 2018), doch die Stabilität der Verteilung der verfügbaren Einkommen ist im Vergleich etwa zu den USA nahezu spektakulär (SVR 2017; Feld und Schmidt 2016).

### Eine kurze Chronologie der Corona-Krise

Wie robust dieses Wirtschaftssystem ist, hat sich in der Corona-Pandemie gezeigt. Im Frühjahr 2020 entwickelte sie sich rasch zu einer weltweiten gesundheitlichen und – vor allem infolge der zu ihrer Abwendung ergriffenen Maßnahmen – wirtschaftlichen Katastrophe. Das Krisenhandeln in Deutschland war zunächst unter großer Unsicherheit über die Wucht der Herausforderung sowie weitgehend ohne Rückgriff auf frühere Präzedenzfälle zu leisten. Zu Beginn des Jahres 2020 bot der erste flächendeckende Lockdown die einzige Möglichkeit, um für den Aufbau einer Strategie des Krisenmanagements Zeit zu gewinnen (acatech 2020).

Dementsprechend wurde von März bis Mai 2020 das gesellschaftliche und wirtschaftliche Leben hierzulande rasant heruntergefahren. Dies hat zu einem starken Einbruch der Wirtschaftsleistung geführt: Im zweiten Quartal 2020 ging das Bruttoinlandsprodukt (BIP) um fast 10 % zurück. Der Tiefpunkt war Mitte Mai 2020 durchschritten. Seitdem hat sich die Wirtschaftsleistung zwar erholt, aber im Schnitt bleibt als Jahresbilanz ein Schrumpfen der Wirtschaft um nahezu 5 %, und die Rückkehr zum ursprünglichen Wachstumspfad dürfte noch geraume Zeit dauern (durchgehend für diesen Abschnitt: GD 2021; SVR 2020).

Im Gegensatz zu regulären konjunkturellen Krisen traf die Corona-Krise zugleich die Angebots- und die Nachfrageseite der Volkswirtschaft. Es kam zugleich zu einer nachfrageseitig bedingten Unterauslastung des Produktionspotenzials, beispielsweise wegen der Sorge um Ansteckungsrisiken am Ort des Konsums und aufgrund von Einkommensverlusten, und zu angebotsseitigen Störungen. Dazu zählen etwa die Unterbrechung international diversifizierter Lieferketten oder Ausfälle in der Belegschaft. Mittelbar entscheidend für den Wirtschaftseinbruch war jedoch der staatliche Eingriff der erzwungenen sozialen Distanzierung.

Schon nach kurzer Zeit führte die enorme Anpassungsfähigkeit der privaten Wirtschaft zu umfangreichen Anpassungen der Produktionsprozesse, etwa durch Schichtbetrieb oder Homeoffice-Regelungen, des Produktportfolios und der Prozesse der Leistungserbringung, insbesondere durch den Aufbau von Lieferservices. Im Verlauf des Jahres wurden dann in unvorstellbarer Geschwindigkeit Impfstoffe gegen den neuen Erreger entwickelt und der Genehmigung zugeführt. Gleichzeitig schulterten Familien, insbesondere solche mit schulpflichtigen Kindern und pflegebedürftigen Angehörigen, eine gewaltige Anpassungslast.

Um die Konsequenzen für Wirtschaftsakteure abzufedern, haben Bund und Länder eine Vielzahl von Maßnahmen ergriffen. Sie reichten von Liquiditätshilfen durch Kredite und Transfers bis zur Kurzarbeit, um Arbeitsstellen zu erhalten. Dies war im Rahmen der Schuldenbremse aufgrund der Sonderregelung für gesamtwirtschaftliche Notfälle möglich. Die Politik hat somit viel getan, um die negativen Konsequenzen der Pandemie zu begrenzen. Weil die Haushaltspolitik der vergangenen Jahre erhebliche Handlungsspielräume geschaffen hatte, konnte die fiskalische Reaktion Deutschlands recht stark ausfallen.

Gleichzeitig schaltete das Regierungshandeln von Bund, der Ländern und Kommunen erkennbar in einen kräftezehrenden Krisenmodus. Allerdings kann die Erholung der Wirtschaftsleistung nach der Rücknahme des Lockdowns im Sommer 2020 wohl kaum allein den vielfältigen staatlichen Maßnahmen zugerechnet werden. Sie wäre ohne dieselben vermutlich weniger stark ausgefallen, aber ausgeblieben wäre sie nicht: Die Gewährung unternehmerischer Freiheit führt direkt zu einer Revitalisierung der Wirtschaft. Insgesamt legt der internationale Vergleich nahe, dass die deutsche Volkswirtschaft diese Krise bislang recht gut gemeistert hat.

In einigen Schlüsselbereichen staatlichen Handelns wurden die Prozesse der Leistungserbringung jedoch offenbar nicht annähernd mit der gleichen Konsequenz angepasst wie in der privaten Wirtschaft. Mit der Einsicht der Rückschau ausgestattet, wissen wir heute, dass im Schulwesen und im öffentlichen Gesundheitswesen das deutliche Abklingen der Neuinfektionen ab Mai 2020 und die im Vergleich zu den Anpassungserfordernissen der privaten Wirtschaft und der Familien üppige Zeit bis zum Herbst 2021 hätte genutzt werden müssen, um sich gegen das Auftreten einer zweiten massiven Infektionswelle zu wappnen.

### Eine neue Sensibilität: Wirtschaftsleistung schafft Handlungsspielräume

Die Geschwindigkeit, mit der sich die Corona-Pandemie zu einer weltumspannenden Katastrophe ausgewachsen hat, sollte eigentlich unsere Sinne für die Vulnerabilität unseres Wohlstands neu geschärft haben. Sie steht in starkem Kontrast zu jenem vergleichsweise gemächlichen Tempo, mit dem andere große Herausforderungen, allen voran die demographische Alterung und die anthropogen verursachte Erderwärmung, im täglichen Leben wirksam werden. Der binnen weniger Monate erlebte Absturz der Wirtschaftsaktivität hat deutlich gezeigt, wie fragil unser materieller Wohlstand ist.

Vor allem muss die Wirtschaftsleistung in jedem Jahr von Grund auf neu erarbeitet werden, es handelt sich beim Bruttoinlandsprodukt um eine Stromgröße, die zu Jahresanfang bei null startet – aber deren Zeitreihe erfreulicherweise meist eine hohe Persistenz aufweist. In wirtschaftspolitischen Diskussionen dominieren daher in konjunkturellen Normalzeiten statt Fragen zum Niveau solche zur Wachstumsrate des Produktionspotenzials – die aktuell rund 1 % beträgt – und zu möglichen Wegen, diese zu steigern. Und es wird diskutiert, wie sich dazu die tatsächliche Wachstumsrate verhält, also die Konjunktur.

Nun zeigt die Bilanz des Corona-Jahres 2020 unmissverständlich: Ein länger anhaltender Stillstand des Wirtschaftslebens bedeutet einen Wohlstandsverlust. Der Rückgang der Wirtschaftsleistung um rund 5 % wirft die deutsche Volkswirtschaft zwar lediglich auf ein vor wenigen Jahren erlebtes hohes Wohlstandsniveau zurück. Aber der moderate Rückgang im Aggregat vermischt disruptive Erfahrungen, die eine Minderheit mit der Vernichtung unternehmerischer Existenzen und des Arbeitsplatzverlusts durchlebt, mit den Erfahrungen einer Mehrheit in weitgehend stabilen wirtschaftlichen Verhältnissen.

Letztlich kommt es bei der Einschätzung der langfristigen Leistungsfähigkeit von Wirtschaftsordnungen nicht nur darauf an, ob das durchschnittliche Mitglied einer Gesellschaft ein hohes Wachstum der materiellen Prosperität erlebt. Vielmehr geht es auch darum, ob dabei eine kohärente Verteilung von wirtschaftlichen Handlungsoptionen und Lebenschancen realisiert – und ob in einer Krise den Schwächsten wirksam geholfen wird. So sind etwa in den USA, deren Gesellschafts- und Wirtschaftssystem den sozialen Ausgleich weniger stark betont, die Wirtschaftsleistung pro Kopf höher und zugleich die Armut stärker prävalent als hierzulande.

Doch obschon die aktuelle Krise Gelegenheit dazu geboten hätte, die Krisenfestigkeit der hiesigen sozialen Sicherungssysteme zu würdigen, scheint sich die Verteilungsdebatte sogar noch intensiviert zu haben. Vielen ist offenbar nicht präsent, dass Wirtschaftsleistung die Grundlage für Sozialleistungen ist: Die Möglichkeit zur staatlichen Schuldenaufnahme erlaubt es, die aktuelle Wirtschaftsleistung vom Umverteilungspotenzial zu entkoppeln. Das ist zwar hilfreich, um konjunkturelle Schwankungen zu glätten, verstellt jedoch den Blick darauf, dass immer wieder neu erarbeitet werden muss, was zum Zweck des sozialen Ausgleichs verteilt wird.

### Der Weg zurück zum Prosperitätswachstum

Selbst wenn die Corona-Pandemie im Laufe des Jahres 2021 erfolgreich überwunden werden sollte, muss stabiles Prosperitätswachstum erst wieder neu gesichert werden. Schon vor der Corona-Krise standen Wirtschaft und Gesellschaft in Deutschland vor einem tiefgreifenden Strukturwandel (SVR 2019b). In den 2020er Jahren werden die geburtenstarken Jahrgänge der 1950er und 1960er-Jahre verstärkt ins Rentenalter eintreten. Die Erwerbsbevölkerung wird infolgedessen schrumpfen, und Fachkräfteengpässe werden sich verschärfen. Es dürfte unrealistisch sein, diesen Impuls allein durch Zuwanderung abfedern zu wollen (SVR 2018).

Eine alternde Bevölkerung könnte in der Tendenz zu einem weiteren Rückgang der Innovationsdynamik führen und die Attraktivität Deutschlands als Investitionsstandort weiter schmälern. Dabei hatte die deutsche Volkswirtschaft schon seit vielen Jahren sinkende Produktivitätszuwächse aufgewiesen (SVR 2019b, 2020). Nun hat die Pandemie eine eigene Veränderungsdynamik hervorgerufen. So ist nicht ausgeschlossen, dass es aufgrund der langanhaltenden wirtschaftlichen Durststrecke im Nachgang zur Krise zu einem nennenswerten Anstieg von bislang durch Stützungsmaßnahmen verhinderten Insolvenzen kommen wird.

Dieses verschärfte Ausscheiden aus dem Marktprozess könnte eine unerwünschte Parallele in einer gebremsten Gründungsdynamik finden. Wie ernsthaft unternehmerische Existenzrisiken zu wägen sind, hat sich in der aktuellen Krise schließlich deutlich gezeigt. Auch das vor Ort vorhandene Humanvermögen, das bislang einen wichtigen Vorteil des Investitionsstandorts Deutschland dargestellt hatte, könnte in der Krise erheblichen Schaden genommen haben. Abzuwarten bleibt zudem, wie gut sich der Staat künftig wieder aus der in der Krise gewachsenen Erwartungshaltung befreien kann, Lebensrisiken nahezu vollständig abzufedern.

Darüber hinaus hat die Pandemie selbst eigene Impulse für Strukturwandel in Gang gesetzt. So hat sich die Digitalisierung der Wirtschaft rasant beschleunigt und so zum einen neue Geschäftsmodelle ermöglicht und zum anderen bisherige Geschäftsmodelle ihrer Rentabilität beraubt, etwa im Transport- und Beherbergungsgewerbe. Vor allem sind erhebliche Lücken bei der Digitalisierung des staatlichen Verwaltungshandelns und im Bildungsbereich offenbar geworden, die nicht allein durch einen – ebenso notwendigen – Ausbau der digitalen Infrastruktur allein geschlossen werden können (SVR 2018).

Zudem hat die Pandemie verdeutlicht, wie verletzlich weltumspannend ausdifferenzierte Lieferketten sind, sodass Unternehmen – und letztlich auch ganze Staaten – nach einer neuen Balance zwischen wirtschaftlicher Effizienz und Widerstandsfähigkeit suchen müssen (Kagermann et al. 2021b). Dieser Verletzlichkeit abzuhelfen, liegt in erster Linie in der Verantwortung der Unternehmen selbst. Ein lenkender Eingriff des Staates lässt sich nur dann rechtfertigen, wenn die privaten Interessen der Unternehmen an der Sicherstellung ihrer Funktionsfähigkeit von den gesellschaftlichen Interessen einer Absicherung gegen Krisen abweichen.

Vor diesem Hintergrund stellt sich die Frage, welche grundlegende Ausrichtung der Wirtschaftspolitik am besten dazu geeignet wäre, diesen anstehenden massiven Strukturwandel zu ermöglichen und voranzutreiben. Den Vorstellungen der Sozialen Marktwirtschaft zufolge ist dazu eine Wirtschaftspolitik erforderlich, die vorwiegend als Standort- und Innovationspolitik ausgerichtet ist, indem sie gute Rahmenbedingungen für unternehmerisches Handeln und einen dynamischen Strukturwandel bietet, und die zugleich die Anpassungslasten der vom Strukturwandel negativ Betroffenen abfedert (SVR 2019b).

Aktivitäten im Bereich von Forschung, Innovation und Wissenstransfer gehen typischerweise mit positiven externen Effekten einher und sollten daher staatlich gefördert werden. Der in der Sozialen Marktwirtschaft bewährte „horizontale“ Ansatz der Förderung nimmt davon Abstand, einzelne Technologien, Unternehmen oder Sektoren zu bevorzugen. Insbesondere in Fällen von technologiespezifischem Markt- oder Koordinationsversagen kann zwar die „vertikale“ Förderung einzelner Sektoren oder Technologien sinnvoll sein, sollte aber an hohe Anforderungen an wettbewerbliche Vergabe und Evaluierung gebunden sein (SVR 2019b).

Dieser agnostische industriepolitische Ansatz wurde bereits vor Ausbruch der Corona-Krise kritisch hinterfragt, da einige Volkswirtschaften immer deutlicher erkennbar versuchen, ihren Unternehmen durch gezielte Förderung Vorteile im internationalen Wettbewerb zu verschaffen (BMWi 2019). Insbesondere die in der Corona-Krise offenbar gewordene Bedeutung von Impfstoffen und medizinischem Material hat nun dem Werben für den Einsatz einer eigenen strategisch orientierten Industriepolitik und dem Streben nach Technologiesouveränität noch weiteren Rückenwind verliehen (Kagermann et al. 2021a; Streibich und Lenarz 2021).

Die Soziale Marktwirtschaft besitzt hinreichende Bodenhaftung, um zu erkennen, dass es tatsächlich Fragen von „strategischer Bedeutung“ gibt, bei denen eine edle Zurückhaltung des Staates das gesellschaftliche Interesse empfindlich bedrohen könnte. Dies dürfte bei Querschnittstechnologien der Fall sein, die breite Ausstrahlungseffekte auf die wirtschaftliche Aktivität haben. Sie ruht allerdings ebenso fest auf der Einsicht, dass ein kompetenter Staat wachsam eingreifen muss, um nicht unter dem Deckmantel des gesellschaftlichen Interesses für die Sicherung von Einzelinteressen vereinnahmt zu werden (SVR 2019b).

## Ökologische Erneuerung der Sozialen Marktwirtschaft

Die Aufgabe, bis zur Mitte des Jahrhunderts in Europa für den Umstieg auf ein klimaneutrales Energiesystem – die in Europa verursachten Emissionen und der Atmosphäre entzogenen Treibhausgase liegen bilanziell bei null – zu sorgen, erzwingt eine grundlegende Transformation des Energiesystems. Dieser Umbau muss weg von fossilen, hin zu erneuerbaren Energieträgern führen. Eine derartige „Defossilisierung“ muss zwar in Europa gelingen, aber um der Erderwärmung wirksam entgegenzuwirken, muss dies möglichst rasch auch global verwirklicht werden (Leopoldina et al. 2020; SVR 2019a).

Für das Schmieden einer globalen Allianz gegen den Klimawandel gibt es keinen eindeutigen Pfad. Letztlich kommt es nur darauf an, dass die insgesamt global emittierte Menge an Treibhausgasen dasjenige Restbudget an Emissionen nicht übertrifft, das mit der von der Weltgemeinschaft angestrebten Beschränkung der globalen Erwärmung auf unter 2 bzw. 1,5 Grad kompatibel ist. Da die Umstellung der Energiesysteme Zeit erfordert, ist es umso schwerer, eine ambitionierte Zielsetzung zu erreichen, wenn man damit erst später anfängt. Daher sind bereits heute ambitionierte klimapolitische Maßnahmen dringlich (Leopoldina et al. 2020).

Doch kann – oder sollte – der deutsche und europäische Beitrag zu dieser nachhaltigen, sektorübergreifenden Transformation zur Klimaneutralität im Rahmen unserer Gesellschafts- und Wirtschaftsordnung gestaltet werden? Oder brauchen wir auch für diese eine „große Transformation“? Diese Fragen ergeben sich zum einen aus der Anforderung, den umfassenden Umstieg in der gebotenen Geschwindigkeit zu bewerkstelligen: Kann dies, so fragen manche, in einem marktwirtschaftlichen System, das individuelle Handlungsfreiheit und private Eigentumsrechte achtet, überhaupt gelingen?

Zum anderen spiegeln sich in diesen Fragen unterschiedliche Wertschätzungen für die Grundpfeiler einer freiheitlichen Gesellschaft: Funktionierende Ökosysteme sind fraglos eine unverzichtbare Basis der menschlichen Existenz, aber gilt es nicht, entlang des Weges dorthin die Errungenschaften der liberalen Gesellschaft, nicht zuletzt die weltweit zunehmend gelingende Befreiung der Menschheit aus materieller Armut, zu bewahren? Denn auch die Transformation zur Klimaneutralität muss selbst nachhaltig gestaltet werden, um gesellschaftlich breite Akzeptanz und vertragstreue internationale Kooperation sicherzustellen (SVR 2019a).

### Globale Defossilisierung: Konzeptionelle Grundlagen

In jedem Falle kann eine Defossilisierung des Energiesystems ohne entschlossenes und tiefreichendes Eingreifen des Staates nicht gelingen. Denn es gilt, ein gewaltiges Dilemma aufzulösen: Die Atmosphäre wird bislang ohne hinreichende Beschränkung als Deponieraum für Treibhausgase genutzt. Denn es handelt sich dabei um ein globales Gemeinschaftsgut, an dem es keine individuellen Eigentumsrechte gibt. Daher sind geeignete Wege staatlichen Eingreifens und der Kooperation zwischen Staaten zu finden, die den Ausstoß von Treibhausgasen begrenzen – und zwar auf der globalen Ebene (Ockenfels und Schmidt 2019).

Den globalen Staat gibt es dabei natürlich nicht, aber eine Gemeinschaft der Nationen hat sich im Abkommen von Paris 2015 darauf verständigt, die Erderwärmung zu begrenzen. Die unmittelbaren Auswirkungen des Klimawandels werden vor allem in den Entwicklungs- und Schwellenländern zu spüren sein, die vergleichsweise geringere Mittel zur Verfügung haben, um sich daran anzupassen. Aus der Perspektive Europas muss es das ausdrückliche Ziel sein, mit der eigenen Transformation zur Klimaneutralität wirksam dazu beizutragen, dass globale Klimaneutralität erreicht wird (Leopoldina et al. 2020).

Eine kohärente europäische Strategie für den Klimaschutz besteht somit aus zwei Kernelementen, die weit über die Festschreibung eigener ambitionierter Ziele hinausreicht. Sie etabliert erstens eine Vorgehensweise, die dazu geeignet ist, auf globaler Ebene als Vorbild zu dienen. Dies erfordert natürlich erfolgreiches klimapolitisches Handeln. Aber im Gegensatz zu einer Rolle als Vorreiter strebt sie dabei nicht entlang des Weges immer ambitioniertere Ziele an, ohne dafür von anderen Volkswirtschaften ebenfalls hohe Ambitionen als Gegenleistung einzufordern (SVR 2019a).

Die eigenen klimapolitischen Ziele wirksam zu erreichen, ist lediglich die Minimalanforderung. Diese ließen sich schließlich auch unter massivem Wohlfahrtsverlust durch die Reduktion der Wirtschaftsleistung verwirklichen. Als Vorbild für andere Volkswirtschaften könnte Europa so aber nicht dienen. Vielmehr muss die angestrebte Rückführung der Emissionen unter (i) möglichst geringem Einsatz von volkswirtschaftlichen Ressourcen, (ii) Sicherstellung sozialer Ausgewogenheit und (iii) Wahrung der internationalen Wettbewerbsfähigkeit der heimischen Unternehmen gelingen (SVR 2019a).

Eine kohärente europäische Strategie ließe zweitens nichts unversucht, die Bildung einer globalen Allianz für den Klimaschutz voranzutreiben. Auf globaler Ebene als Vorbild zu wirken, ist zwar unverzichtbar, aber es genügt nicht. Vielmehr gilt es, in internationalen Verhandlungen andere Volkswirtschaften unnachgiebig zu entsprechenden Zugeständnissen – etwa die Erhebung eines CO_2_-Preises – zu drängen, aber zugleich als eigene Gegenleistung für diese Zugeständnisse Hilfen bei der Anpassung an die Folgen des Klimawandels oder andere Transfers in Aussicht zu stellen (Ockenfels und Schmidt 2019; SVR 2019a).

### Rationale Energiewendepolitik: Effiziente Koordination

Die Anforderung der wirtschaftlichen Effizienz hat eine klare Implikation: Die Transformation ist ausdrücklich als eine sektorübergreifende Aufgabe zu verstehen, bei der es in allererster Linie auf das Gesamtergebnis bei der Entwicklung der europäischen Emissionen ankommt und nicht auf die Entwicklungen in einzelnen Sektoren. Auf dem Weg zur Klimaneutralität sind sektorale Entwicklungen nur in dem Maße bedeutsam, in dem ein Zurückbleiben bei der Reduktionsleistung aufgrund von Pfadabhängigkeiten und langfristigen Investitionszyklen das Erreichen des Gesamtziels erschwert (durchgehend für diesen Abschnitt: SVR 2019a).

Auf dem Pfad zur Klimaneutralität variieren die Kosten für die Vermeidung der jeweils nächsten Tonne an Treibhausgasemissionen zwischen Sektoren, Regionen und Akteuren. Es liegt nahe, dass diese Kosten in der Industrie und in der Energiewirtschaft tendenziell am geringsten sind. In den Bereichen Verkehr und Wärme dürften die Vermeidungskosten höher sein. Die genaue Verteilung dieser Kosten über alle relevanten Akteure hinweg ist aber nicht bekannt, da in diesen Bereichen nur wenig auf Erfahrungswerte zurückgegriffen werden kann. Letztlich sind sie nur auf dem Weg von Versuch und Irrtum empirisch zu ermitteln.

Informationsdefizite sprechen gegen stark einen planwirtschaftlichen Ansatz für den Weg zur Klimaneutralität. Nur wenn die individuellen Vermeidungskosten bekannt wären, ließe sich ein detaillierter volkswirtschaftlich effizienter Plan entwerfen, um zunächst die Vermeidungsoptionen mit den geringsten Kosten zu verwirklichen und darauf zu setzen, dass durch technischen Fortschritt immer wieder neue kostengünstige Vermeidungsoptionen verfügbar werden. Eine intelligente – überwiegend, aber nicht ausschließlich „horizontale“ (s. oben) – Innovationspolitik ist unabhängig vom gewählten Koordinierungsmechanismus sinnvoll.

Gute Argumente sprechen hingegen für einen marktwirtschaftlichen Ansatz. Die Logik einer möglichst intensiven Arbeitsteilung bei der Transformation spricht für die Erhebung eines über alle Sektoren einheitlichen und im Zeitablauf steigenden CO_2_-Preises. Denn Preissignale können die Handlungen einer großen Zahl von Akteuren koordinieren, ohne diese physisch zusammenzuführen – und ohne ihnen ihr Verhalten und ihre Entscheidungen vorzuschreiben: Ein CO_2_-Preis sorgt dadurch für einen Umstieg, dass die Nutzung fossiler Energieträger weniger attraktiv wird als ihre Alternativen.

Verwirklichen lässt sich eine Bepreisung von Emissionen entweder durch die Festlegung von handelbaren Emissionsrechten („Mengensteuerung“), deren jährlich verfügbare Menge im Einklang mit dem angestrebten Reduktionspfad für die Emissionen schrittweise zurückgenommen wird. Der CO_2_-Preis ergibt sich dabei als Marktergebnis und reflektiert tendenziell eben jene Vermeidungskosten, die gerade bei der Vermeidung der jeweils letzten Einheit an Treibhausgasen zur Einhaltung der aktuellen Obergrenze anfallen – ohne Anschauung des dabei betroffenen Akteurs.

Oder der Gesetzgeber erhebt eine Steuer auf die Nutzung fossiler Energieträger, die deren Emissionsgehalt entspricht, und wählt deren Pfad so, dass der angestrebte Reduktionspfad verwirklicht wird („Preissteuerung“). Bei einer Mengensteuerung müssen hohe CO_2_-Preise, sollten sie sich als Marktergebnis einstellen, politisch durchgehalten werden, bei einer Preissteuerung muss der politische Wille vorhanden sein, die CO_2_-Steuer anzuheben, wenn der angestrebte Vermeidungspfad nicht erreicht wird. Denn es kann nicht gelingen, auf Anhieb den „richtigen“ CO_2_-Preis festzulegen.

Ohnehin geht es in beiden Fällen darum, ein in seiner Dauerhaftigkeit glaubwürdiges System der Bepreisung einzurichten. Denn Unternehmen und Haushalte handeln bei der Investition in Produktionsanlagen und Maschinen sowie beim Erwerb langfristiger Haushaltsgüter vorausschauend. Sie beziehen typischerweise ihre Erwartungen über künftige Veränderungen der Rahmenbedingungen in die aktuellen Entscheidungen ein. In der Konsequenz sind nicht nur die aktuellen Anreize, sondern auch die verlässliche Gestaltung dieser Rahmenbedingungen von hoher Bedeutung.

Natürlich reicht es nicht aus, allein auf CO_2_-Preise zu setzen. In einer Marktwirtschaft hat die öffentliche Hand eine wichtige Rolle beim Beheben von Markt- und Koordinationsversagen, insbesondere durch die Bereitstellung einer hochwertigen Infrastruktur und die Gewährleistung eines fairen Wettbewerbs. Dies gilt auch bei einer marktwirtschaftlichen Organisation der Energiewende. In ihrem Zentrum sollte ein CO_2_-Preis als Leitinstrument, nicht als Solist, stehen, dessen Funktionieren der Staat – ganz im Sinne der Sozialen Marktwirtschaft – durch flankierendes Handeln sichert.

Ein CO_2_-Preis macht die dabei entstehenden individuellen Belastungen zwar transparent, aber er ist nur der Bote der Erkenntnis, dass eine umfassende Transformation des Energiesystems nicht umsonst zu haben ist. Planwirtschaftliche Vorgaben für die Handlungen einzelner Akteure verfehlen insbesondere dann das Ziel, für ein volkswirtschaftlich effizientes Ergebnis zu sorgen, wenn die Informationslage komplex ist. Bei der Energiewende ist dies wegen der schier unüberschaubaren Anzahl von Akteuren des Energiesystems und der Dynamik technologischer Entwicklungen in besonderem Maße der Fall.

Insgesamt drängt sich somit eine marktwirtschaftliche Organisation der Energiewende als Vorgehensweise nahezu auf: Aufgrund der Fähigkeit von Märkten, die Entscheidungen, Vorlieben und Einstellungen vielfältiger Akteure so zu koordinieren, dass ein vorgegebenes Ziel – hier die Rückführung von Treibhausgasemissionen – zu den geringstmöglichen Kosten verwirklicht wird, wird eine marktwirtschaftlich organisierte Transformation zur Klimaneutralität vergleichsweise geringe volkswirtschaftliche Kosten aufwerfen. Aber gewisse Kosten sind bei diesem Umstieg unvermeidlich.

Bislang verfolgen die deutsche und die europäische Energiewendepolitik stattdessen einen Ansatz des „viel hilft viel“, bei dem eine Fülle einander überlappender Zielvorgaben für einzelne Mitgliedstaaten, Sektoren und Technologien und eine Vielzahl von Instrumenten eingesetzt werden. Doch die Verengung auf die Reduktionsleistungen einzelner Mitgliedstaaten und Sektoren widerspricht dem Prinzip der Arbeitsteilung. Ebenso verkennt die Überhöhung von Effizienzstandards und des Kapazitätsaufbaus bei erneuerbaren Energien zu Zielen mit eigenem Wert ihre Natur als mögliche Instrumente.

Dies alles hat dazu geführt, dass die Reduktionsziele bislang lediglich in demjenigen Bereich eingehalten werden konnten, in dem ein europaweiter Marktmechanismus wirkt, dem europäischen Emissionshandelssystem (EU-ETS). Angesichts des nun deutlich gestiegenen Ambitionsgrads wird dieser Ansatz der kleinteiligen Planung aber endgültig an seine Grenzen stoßen. Um die Kosten der Umstellung zu begrenzen, gilt es, die bisherige nationale und sektorale Verengung zu überwinden und durch die Ausweitung des EU-ETS auf alle relevanten Bereiche auf eine marktwirtschaftliche Koordination umzustellen.

### Gesellschaftliche und wirtschaftliche Einbettung

Es geht bei der Transformation zur Klimaneutralität aber nicht allein um die Qualität unterschiedlicher Ordnungssysteme bei der Eindämmung der Umstellungskosten. Vielmehr muss der Umstieg auch gesellschaftlich eingebettet sein, wenn das Vorhaben nicht am Widerstand der dezentralen Akteure scheitern soll. Es steht dabei nicht weniger auf dem Spiel als seine Gelingensbedingungen in der realen Welt. Appelle an die moralische Qualität des individuellen Handelns werden dazu nicht ausreichen. Vielmehr geht es darum, eine erkennbar fair gestaltete Verteilung der Anpassungslasten zu gewährleisten (Preuss et al. 2019).

Werden keine staatlichen Maßnahmen der Kompensation ergriffen, führt Klimapolitik unweigerlich zu Verteilungswirkungen. Dies gilt gleichermaßen für preisliche wie nicht-preisliche Instrumente. Dabei werden einkommensschwache Haushalte relativ zu ihrem Einkommen stärker belastet, weil sie einen größeren Anteil ihres Budgets für die Nutzung von Energie einsetzen. Eine CO_2_-Bepreisung ist aus sozialpolitischer Sicht vorteilhaft, weil sie die Gesamtkosten des Umstiegs begrenzt und Mittel für die Kompensation einsammelt.

Ein rasch ansteigender CO_2_-Preis kann die Wettbewerbsfähigkeit europäischer Unternehmen gegenüber ihren globalen Konkurrenten beeinträchtigen und droht daher zur Verlagerung der Produktion und an Standorte außerhalb Europas zu führen. Ein solcher „Carbon Leakage“ wäre nicht nur schädlich für das globale Klima, sondern würde auch Arbeitsplätze und wirtschaftlichen Wohlstand in Europa gefährden. Bisher konnten diese negativen Konsequenzen im Rahmen des EU-Emissionshandels durch die teilweise kostenlose Zuteilung von Emissionszertifikaten an energieintensive Industrieunternehmen vermieden werden (FGCEE 2021).

Die im Rahmen des European Green Deal verschärften Klimaziele machen nun deutlich höhere CO_2_-Preise erforderlich, sodass die Drohung möglicher Produktionsverlagerungen an Virulenz gewinnen wird. Ein Versuch, um den heimischen Unternehmen faire Wettbewerbsbedingungen zu sichern, wäre die Einrichtung eines CO_2_-Grenzausgleichs: Eine nach dem impliziten CO_2_-Gehalt importierter Güter bemessene Abgabe würde alle in der EU genutzten Produkte mit dem gleichen CO_2_-Preis belasten. Da Exporte aber nicht entlastet würden, entsteht ein Dilemma zwischen globalem Klimaschutz und europäischer Wettbewerbsfähigkeit.

Der einzige Ausweg, um dieses Dilemma aufzulösen, besteht im Schmieden breiter internationaler Allianzen für einen marktwirtschaftlich organisierten Klimaschutz. Zum einen wird es der EU leichter fallen, dafür zu werben, wenn sie selbst diesen Weg erfolgreich beschreitet. Zum anderen sollte die EU die eigene Regeltreue und Vorbildlichkeit bei der Umsetzung der globalen Vertragswerke mit einer hohen Bereitschaft zum Transfer von Finanzressourcen und Technologien verbinden. Nur dann wird es voraussichtlich für aufstrebende Nationen hinreichend attraktiv sein, dieser Allianz beizutreten.

Diese Bereitschaft wird nicht zuletzt auch dringend benötigt, um dem globalen Machtanspruch Chinas entgegenzuwirken und im damit verbundenen Systemwettbewerb europäischen Wertvorstellungen von Freiheit, Demokratie, Aufklärung, Subsidiarität und Solidarität Gewicht zu verleihen. Nur ein wirtschaftlich erfolgreiches Europa wird diesen Anspruch auf globaler Ebene durchsetzen können. Damit dies gelingt, sollte Europa weiterhin selbstbewusst die prägende Wirtschafts- und Gesellschaftsordnung der Sozialen Marktwirtschaft vertreten und auf die hier geschilderte ökonomisch rationale Weise um ökologische Inhalte erweitern.
